# The Role of Corpus Callosum Development in Functional Connectivity and Cognitive Processing

**DOI:** 10.1371/journal.pone.0039804

**Published:** 2012-08-03

**Authors:** Leighton B. N. Hinkley, Elysa J. Marco, Anne M. Findlay, Susanne Honma, Rita J. Jeremy, Zoe Strominger, Polina Bukshpun, Mari Wakahiro, Warren S. Brown, Lynn K. Paul, A. James Barkovich, Pratik Mukherjee, Srikantan S. Nagarajan, Elliott H. Sherr

**Affiliations:** 1 Department of Radiology and Biomedical Imaging, University of California San Francisco, San Francisco, California, United States of America; 2 Department of Neurology, University of California San Francisco, San Francisco, California, United States of America; 3 Department of Pediatrics, University of California San Francisco, San Francisco, California, United States of America; 4 Travis Research Institute, Fuller Theological Seminary, Pasadena, California, United States of America; 5 Department of Neuroscience, Caltech, Pasadena, California, United States of America; University Medical Center Groningen UMCG, The Netherlands

## Abstract

The corpus callosum is hypothesized to play a fundamental role in integrating information and mediating complex behaviors. Here, we demonstrate that lack of normal callosal development can lead to deficits in functional connectivity that are related to impairments in specific cognitive domains. We examined resting-state functional connectivity in individuals with agenesis of the corpus callosum (AgCC) and matched controls using magnetoencephalographic imaging (MEG-I) of coherence in the alpha (8–12 Hz), beta (12–30 Hz) and gamma (30–55 Hz) bands. Global connectivity (GC) was defined as synchronization between a region and the rest of the brain. In AgCC individuals, alpha band GC was significantly reduced in the dorsolateral pre-frontal (DLPFC), posterior parietal (PPC) and parieto-occipital cortices (PO). No significant differences in GC were seen in either the beta or gamma bands. We also explored the hypothesis that, in AgCC, this regional reduction in functional connectivity is explained primarily by a specific reduction in interhemispheric connectivity. However, our data suggest that reduced connectivity in these regions is driven by faulty coupling in both inter- and intrahemispheric connectivity. We also assessed whether the degree of connectivity correlated with behavioral performance, focusing on cognitive measures known to be impaired in AgCC individuals. Neuropsychological measures of verbal processing speed were significantly correlated with resting-state functional connectivity of the left medial and superior temporal lobe in AgCC participants. Connectivity of DLPFC correlated strongly with performance on the Tower of London in the AgCC cohort. These findings indicate that the abnormal callosal development produces salient but selective (alpha band only) resting-state functional connectivity disruptions that correlate with cognitive impairment. Understanding the relationship between impoverished functional connectivity and cognition is a key step in identifying the neural mechanisms of language and executive dysfunction in common neurodevelopmental and psychiatric disorders where disruptions of callosal development are consistently identified.

## Introduction

Consisting of over 190 million axons, the corpus callosum is the largest white matter tract and is hypothesized to play a primary role in cognition [1]–[7]. For example emerging evidence suggests that weakened integrity of the callosum directly contributes to a decline in cognitive function in aging adults [8], [9] whereas increased callosal thickness in typical childhood development correlates with intelligence [10]–[12], processing speed [13] and problem solving abilities [14]. There is also a growing body of literature reporting that subtle structural changes in the corpus callosum may correlate with cognitive and behavioral deficits in neurodevelopmental disorders including autism [15], [16], schizophrenia [17]–[21] and attention-deficit disorder [22]. It has been hypothesized that both executive function and social cognition deficits seen in these patients may be unified by abnormal callosal development and function [23]. Furthermore, there are numerous examples of genetic processes that link autism, schizophrenia and disrupted callosal development, such as mutations in the genes ARX [24] and DISC1 [25] and copy number variants in the 16p11.2 locus [26], [27].

Individuals with agenesis of the corpus callosum (AgCC), including those with complete (cAgCC) and partial (pAgCC) absence of the corpus callosum, present a unique opportunity for understanding the relationship between the development of callosal integrity and cognitive processing. For instance, there is emerging evidence indicating that even in individuals in the normal IQ range, anatomically overt abnormalities of callosal development (including its complete absence) lead to deficits in cognitive abilities. Primarily, individuals with AgCC show deficits in both problem solving abilities [28], [29] and processing speed [30]. Preliminary evidence suggests that impairments in domains such as abstract reasoning [31], [32], verbal fluency and second-order linguistic deficits [7], [31], [33] as well as social cognition [34]–[36] may indeed be secondary to core problem solving and processing speed deficits in these individuals [32], [37].

Collectively, these findings suggest that an intact corpus callosum contributes significantly to efficient executive function, or the execution and maintenance more generally of complex cognitive abilities [38]. However, it is not clear if the failure of corpus callosum development simply disrupts interhemispheric coupling or if there is a selective vulnerability in callosal dysgenesis to certain cognitive domains that are dependent on efficient interhemispheric communication. Moreover, because the underlying disruptions in brain development and cerebral connectivity that lead to AgCC may also alter intrahemispheric connectivity, it is possible that AgCC individuals have additional cognitive deficits that are caused by impairment of intrahemispheric connectivity. Our prior work showing that the cingulum bundle is smaller and has a lower fractional anisotropy in AgCC individuals supports this more nuanced hypothesis [39]. The study of individuals with AgCC informs our understanding of how structural connectivity across the hemispheres contributes to other, more broadly defined, neurodevelopmental disorders thought to result from decreased long-range interactions in the brain [16], [23], [40].

To address these questions, we used magnetoencephalography (MEG) to examine resting state functional connectivity in AgCC individuals and age- and IQ-matched healthy controls. MEG permits the imaging of brain activity in the sub-millisecond time-scale by measuring magnetic field changes on or near the scalp surface. Whole brain activity can be estimated from MEG sensor data using source reconstruction algorithms that allow one to overlay cortical oscillatory activity onto subjects' anatomical images in order to localize changes in cortical oscillations to specific brain regions. This process is referred to as magnetoencephalographic imaging (MEG-I). Advances in statistical signal processing have improved the spatiotemporal resolution of MEG-I and have also more recently been adapted to interrogate functional interactions in cortical oscillatory activity across brain regions and frequency ranges [41]–[43]. This study capitalizes upon these advances by using MEG-I and novel methods for assessing functional connectivity to address how callosal dysgenesis in AgCC contributes to compromised cognitive processing.

In the present study, we measured MEG-I resting-state connectivity to test the hypothesis that AgCC individuals have decreased functional connectivity in comparison to age and IQ-matched controls. It has been proposed that ipsilaterally projecting fibers that develop in callosal agenesis patients (i.e. Probst bundles) [44] selectively aid in the within-hemisphere transfer of information in the absence of fully formed cross-hemispheric tracts. Therefore, we further address whether connections within a single hemisphere (intrahemispheric connectivity) are increased as a compensation for diminished cross-hemisphere (interhemispheric) connections. We also hypothesize that individuals with complete AgCC (cAgCC) will have diminished interhemispheric coherence relative to individuals where partial (pAgCC) callosal fragments remain that could serve interhemispheric transfer. Finally, we propose that the degree of resting-state functional connectivity will, in individuals with AgCC, correlate with performance in cognitive domains known to be affected in these individuals: problem solving [32] and verbal processing speed [30].

## Materials and Methods

### Ethics Statement

This study was approved by the UCSF Committee on Human Research, and all experiments were conducted in accordance with the Declaration of Helsinki. All participants gave written informed consent to participate. Additional written consent was obtained from the parents/guardians of the participants involved who were under the age of 18.

### Participants

This study involved 18 patients with agenesis of the corpus callosum (AgCC) aged 17–57 years (mean age 31, SD = 12.1) recruited through the UCSF Comprehensive Center for Brain Development (Tables 1 and 2). Six of the participants were female (30%), four were left handed (22%) and two were ambidextrous (11%). The AgCC cohort had a mean FSIQ of 100 (range 77–129; SD = 13.7). Of the 18 AgCC patients, nine had complete agenesis of the corpus callosum and nine had partial agenesis of the corpus callosum. The FSIQ for pAgCC was 101 (range 77–129; SD = 16.2) and for cAgCC was 98 (range 78–112; SD = 13.7). Eighteen right-handed healthy control (HC) participants (ten female) matched for age and IQ were recruited from the greater San Francisco Bay Area. The mean age for this sample was 29 years (range 19–51 years, S.D. = 11) and the mean FSIQ was 105 (range 89–115, S.D. 7.1.) There was no significant difference between age or FSIQ for the AgCC cohort and controls. Furthermore, there was no significant difference between the pAgCC and cAgCC age cohorts on age or FSIQ (all p>0.05). The diagnosis of AgCC was made based on radiographic review by at least two of these authors based on published criteria (E.H.S, A.J.B. and P.M) [45]. All participants were assessed with a comprehensive research battery, including: medical history, physical and neurological examination, genetic testing, neuropsychological evaluation, and diagnostic MR imaging.

**Table 1:** Complete Agenesis of the Corpus Callosum Cohort Characterization.

ID	VIQ	PIQ	P/C	CC details	PB	AC	HC	CO	WM	Other MRI findings:	Medications	Other diagnosis:
1054	91	82	C	Absent	+	Nl	−	–	Mild	none	Levetiracetam Gabapentin	Shapiro Syndrome
1426	86	91	C	Absent	+	Nl	−	+	Mild	periventricular heterotopia	Escitalopram	ADHD, Depression
1240	83	83	C	Absent	+	Nl	−	+	Mod	none	None	None
1062	99	109	C	Absent	+	Nl	−	+	Mod	perisylvian polymicrogyria & posterior fossa arachnoid cyst	None	Depression & Auditory Hallucinations
1258–5	102	104	C	Absent	+	Lg	−	+	Mild	none	None	None
1198	90	118	C	Absent	+	Nl	−	+	Severe	periventricular heterotopia & right cerebellar dysplasia	None	ASD
1516	105	111	C	Absent	+	Nl	−	+	Mod	abnormal medial temporal cortical folding	None	None
1202	112	109	C	Absent	+	Lg	−	+	Mod	none	None	None
1386	114	107	C	Absent	+	Lg	−	+	Severe/Mod	None	Lamictal, concerta	Asperger's, ADHD, anxiety, depression

Abbreviations: PIQ  =  performance IQ; VIQ  =  verbal IQ; P/C = (P)artial or (C)omplete callosal agenesis; PB = probst bundle; AC = anterior commisure size; HC = presence of hippocampal commisure; CO  =  presence of colpocephaly; WM  =  degree of white matter reduction (most affected area); P = partial; C = complete; +  = present; −  = absent; Sm = small; Nl = normal; Lg = large; n/a = not available/indeterminate.

**Table 2:** Partial Agenesis of the Corpus Callosum Cohort Characterization.

ID	VIQ	PIQ	P/C	CC details	PB	AC	HC	CO	WM	Other MRI findings:	Medications	Other diagnosis:
1096–0	77	109	P	Absent rostrum, superior genu, body and splenium	+	Nl	–	+	Mod	polymicrogyria, periventricular heterotopia, & interhemispheric cyst	Valproic Acid Prozac	ASD; OCD; Epilepsy
1384	110	87	P	dysplastic with absent genu, superior body, and splenium	+	Lg	+	+	Mod	interhemispheric cyst	None	Depression, Migraine
1015	101	107	P	Absent superior genu, body and splenium	+	Sm	+	+	Severe	abnormal medial temporal cortical folding	None	None
1258–0	100	112	P	Absent rostrum, inferior genu, thin anterior body, absent splenium	+	Nl	–	–	Nl	none	None	Migraine
1477	100	96	P	Absent rostrum, superior genu, body, and splenium	+	Lg	–	+	Mild	none	None	Migraine
1419	135	116	P	Absent inferior genu, posterior body and splenium	–	Lg	−	–	Nl	none	None	None
1605	85	73	P	Absence of rostrum, inferior genu hypoplastic, posterior body and splenium absent	–	Nl	n/a	−	Nl	small ventricles	Lithium, Seroquel	Hearing impaired
1746	90	79	P	Absence of posterior body	−	−	+	−	Mild	Large ventricles	None	None
1671	111	114	P	Posterior genu, anterior body present only	+	Lg	−	+	Mild	Large interhemispheric glioma,	None	Tremor

Abbreviations: PIQ  =  performance IQ; VIQ  =  verbal IQ; P/C = (P)artial or (C)omplete callosal agenesis; PB = probst bundle; AC = anterior commisure size; HC = presence of hippocampal commisure; CO  =  presence of colpocephaly; WM  =  degree of white matter reduction (most affected area); P = partial; C = complete; +  = present; −  = absent; Sm = small; Nl = normal; Lg = large; n/a = not available/indeterminate.

### Neuropsychological Testing

Both individuals with AgCC and healthy controls were evaluated with either the Wechsler Adult Intelligence Scale-III (WAIS-III) [46] or the Wechsler Abbreviated Scale of Intelligence (WASI) [47]. All subjects were administered the Delis-Kaplan Executive Function System (D-KEFS), an executive function battery that probes performance in multiple cognitive domains including processing speed and problem solving [48]. All but one of the behavioral evaluations were conducted or supervised by a single developmental psychologist who was not informed of diagnosis (R.J.J.). The behavioral testing on a single AgCC participant was conducted in the laboratory of Dr. Warren Brown. One AgCC participant did not receive a full behavioral evaluation and was excluded from correlation analyses with the behavioral data. Two composite measures from within the D-KEFS were selected *a priori* for correlations with the functional connectivity data: the Combined Color Naming and Word Reading verbal processing speed (VPS) measure from the Color-Word Interference Test (CWIT-VPS) and the Total Achievement problem-solving (PS) measure from the Tower Test (TT-TA). Although a full D-KEFS was obtained in each participant, we chose these measures as they probe domains of cognitive function that, based on ongoing work in our laboratory, appear to be central to observed behavioral impairments in patients with AgCC. The CWIT-VPS score is representative of basic processing speed (CWIT) [48] and this composite measure from the D-KEFS is reported to reflect processing speed deficits in individuals with callosal agenesis [37]. For this analysis, a composite score (sum of age-scaled scores for Color Naming and Word Reading) was examined as a verbal processing speed measure. The Tower Test (TT-TA score) is a modification of the Tower of London task in the D-KEFS designed to probe processes of problem solving including spatial planning and rule learning [37]. Both the CWIT-VPS and TT-TA are age-scaled continuous variables with a mean of 10 and a standard deviation of 3; higher scores denote better performance.

The D-KEFS behavioral measures were analyzed with STATA version 10 using a one-way MANOVA with corpus callosum status (intact, pAgCC, cAgCC) as the independent multi-level variable and the participants' score on the TT-TA and CWIT-VPS as the continuous dependent measures. The one-way MANOVA comparing an overall effect of group (HC, pAgCC, cAgCC) by test (TT-TA and CWIT-VPS) revealed no statistical group differences (Pillai's Trace 0.06, F(4,52) = 0.42, p = 0.7). The group means were lower and variance was greater in the AgCC cohort for both TT-TA (HC: mean = 10.5, SD = 1.58; AgCC: mean = 9.18, SD = 2.96) and CWIT-VPS (HC: mean = 9.61, SD = 1.33; AgCC: mean = 9.06, SD = 2.70) scores when compared to healthy controls. Although we do not observe significant differences in these measures within our sample, in a similar analysis with a larger cohort that included subjects from this study, there are indeed significant differences in problem solving and processing speed in AgCC [30], [32]. Two factors could contribute to our observation of a lack of significant difference in these neuropsychological measures. First, perhaps it is our smaller sample size (n = 18) compared to the larger cohort study (n = 36; [37]). Second, our sample includes more high-functioning individuals who are more tolerant of brain imaging studies leading to higher performance in our sample. Nevertheless, findings from the larger cohort study motivates the choice of examining relationships between these neuropsychological measures and functional connectivity.

### MRI Acquisition

Structural (T1-weighted) anatomical images were acquired for source space reconstruction, data visualization and second-level group analyses. Scanning was performed using a 3.0T GE Trio scanner installed at Surbeck Lab for Advanced Imaging at the UCSF China Basin campus. For each subject, a 3D-FSPGR high-resolution MRI was acquired (160 1mm thick slices; matrix  = 256×256, TE  = 2.2 ms, TR = 7 ms, flip angle  = 15°).

### Magnetoencephalography Recordings

Data were collected from patients and controls using a whole-head biomagnetometer (MEG International Services, Coquitlam, British Columbia, Canada) containing 275 axial gradiometers at a sampling rate of 1200Hz. For registration, coils were placed at the nasion and 1 cm rostral to the left and right preauricular points angled toward the nasion in order to localize the position of the head relative to the sensor array. These points were later co-registered to a high-resolution structural MR image through a linear affine transformation between fiducial points, MEG device coordinates and MRI coordinates (spherical single-shell head model). Scan sessions where head movement exceeded 0.5 cm within a run were discarded and repeated. Participants were lying in a supine position and instructed to remain awake with their eyes closed during a four-minute continuous recording session.

### Data Reconstruction

From the four minute spontaneous (awake) recording session, a single, contiguous 60s artifact-free segment of the MEG data was selected for analysis. The full recording session was not used as nearly all participants had at least one noisy, transient (<60s) segment of the data that was excluded from the analysis. Furthermore, previous investigations have determined that this 60s window provides reliable, consistent power for reconstruction of brain activity from the resting-state MEG data [41]–[43]. Artifact-free segments were identified qualitatively through a visual inspection of the sensor data, where only segments without excessive scatter (signal amplitude >10 pT) due to eyeblink, saccades, head movement or EMG noise were selected for analysis. Source-space reconstructions and functional connectivity metrics were computed using the Nutmeg software suite (http://nutmeg.berkeley.edu). In each subject, a local sphere was generated based on registration between each MEG sensor and the head surface obtained from the subject's T1-weighted structural MRI, resulting in a multiple local-sphere head model [49]. From this head model, a whole-brain, subject-specific lead field (forward kernels), i.e. the expected sensor data for unit source activity in each voxel and orientation, was computed and used for inverse modeling with adaptive spatial filtering and subsequent functional connectivity analysis.

MEG sensor data were downsampled to 600Hz, bandpass filtered (fourth-order Butterworth filter) and reconstructed in source space using a minimum-variance adaptive spatial filtering technique [50]–[51]. This approach provides a power estimate at each element (voxel) derived through a linear combination of a spatial weighting matrix (itself calculated through a forward-field and spatial covariance matrix) with the sensor data matrix. Reconstructions were not isolated to the cortical surface. Instead, source activity was reconstructed in each voxel within the MRI volume, assuming that at each voxel the source is a dipole with unknown orientation using a vector-source field formulation. While voxel power is estimated in each orientation, power estimates were obtained by summing power across all source orientations.

**Figure 1:**
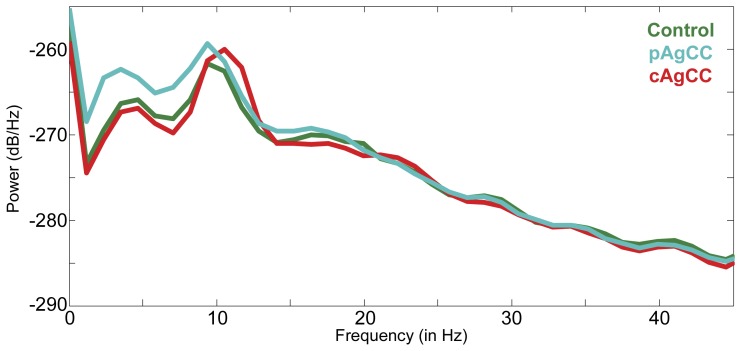
Averaged power spectral density estimates derived from MEG sensors separately for the healthy control (HC; in green), partial (pAgCC; in blue) and complete agenesis of the corpus callosum (cAgCC; in red) groups during a single resting epoch.

For each subject, power spectrograms of this 60s epoch of the sensor data were generated using FFT (1–20 Hz, 1024 samples with a frequency resolution of 1.17 Hz) in order to select a subject-specific alpha band (identified as the greatest power density within the 8–12 Hz range). A peak in the alpha band was easily identifiable from this amount of data in both AgCC patients and controls (Figure 1). Visual inspection of the spectrum revealed no other clearly discernable peaks in the magnetoencephalogram sensor recordings at frequencies greater than 12 Hz. Although peaks in the theta range (4–8 Hz) were discernable in some samples, it was not consistent enough for us to perform subject-specific band selection (as was done for alpha). Therefore, for frequency ranges outside of alpha, we used fixed width frequency bands applied to each subject for our functional connectivity reconstructions in both the patient and control groups for theta (fixed width of 4–8 Hz), beta (fixed width of 12–30 Hz) and gamma (fixed width of 30–55 Hz) frequency bands.

### Global Connectivity Analysis

Functional connectivity was estimated using imaginary coherence (IC), a metric that overcomes estimation biases in MEG source data by isolating non-zero time-lagged interactions [52]. First, we estimate imaginary coherence between two voxels for each frequency within the range of interest (e.g. theta, alpha, beta, gamma). We then take the absolute value of the imaginary coherence across these frequencies within the range of interest and average them to get a since IC value for each voxel pair [41]. Global connectivity (GC) at each location was derived by averaging across all Fisher's Z-transformed IC values between that voxel and all remaining elements (voxels) in the reconstruction. For a second-level group analysis, each individual subject's anatomical T1-weighted MRIs were spatially normalized to the MNI MRI template using standardized procedures [53] in SPM2 (http://www.fil.ion.ucl.ac.uk/spm/software/spm2). This process created a non-linear transformation matrix for each subject that, was then applied to each individual subject's GC map [41], [43]. This non-linear transformation is only used at the group analysis level following source reconstruction of each individual subject's MRI coordinates with subject-specific lead fields, in order to bring all source reconstruction images to a common template (MNI) coordinate system. All the coordinates we report in the paper are in this space.

A comparison between the AgCC connectivity maps relative to HC connectivity was performed using a non-parametric unpaired two-tailed t-test [54]–[55]. Due to the limited sample size in each separate group, voxel-wise comparisons of GC between the pAgCC (n = 9) and cAgCC (n = 9) groups were not conducted.

### Intra/Interhemispheric Connectivity Analyses

A post-hoc analysis to assess within intrahemispheric connectivity and interhemispheric connectivity was conducted using region of interest (ROI) analysis. The ROIs were defined based on the difference in the GC maps of the total AgCC group vs. HC. The euclidian midpoint or center of mass was computed from each significantly different cluster and a seed voxel (20 mm) was generated at this location for each subject. Global functional connectivity (connections between the seed voxel and all other voxels in a given hemisphere) was averaged across each subject for the connections of the ROI within (voxels ipsilateral to ROI) and across (voxels contralateral to ROI) hemispheres. This type of data reduction gives us a single point value (GC average) for a single seed of its connections either within the ipsilateral hemipshere (intrahemispheric connectivity) or between that seed and all other voxels in the contralateral hemisphere (interhemipsheric connectivity). Differences between and within groups were assessed using a 2×2×2 mixed-effects ANOVA varying group (HC vs. AgCC), hemisphere (left vs. right) and connection type (intrahemispheric vs. interhemispheric). Given the reduced sample size (n = 9 in each group) when the AgCC cohort is split into pAgCC and cAgCC subtypes, we also conducted an exploratory planned comparison directly comparing the intra- and interhemsipheric connections between the two groups using two-tailed unpaired t-tests.

### Reliability

In order to derive an estimate of within-session reliability of resting-state functional connectivity analyses using IC, we calculated the intra-class correlation coefficient (ICC; [56]) between two artifact-free, sixty-second epochs within a single functional run. ICCs were computed at each voxel across four frequency bands (theta, alpha, beta, gamma). For the alpha band, good test-retest reliability scores (mean ICC = 0.56) were observed. For the theta and beta bands, fair test-retest reliability scores (theta, mean ICC = 0.36; beta, mean ICC = 0.34) were observed. For the gamma band, poor within-session test-retest reliability was observed (mean ICC = 0.27).

### Behavioral Data Analyses and Functional Connectivity Correlations

To explore the relationship between connectivity and cognition, we did a voxel-wise Pearson's *r* correlation between GC maps and age-scaled scores from the D-KEFS battery. Data was collapsed across pAgCC and cAgCC groups to maximize power in our analyses.

### Multiple Comparisons Correction

For all voxelwise fcMEG-I analyses (including both the whole-brain group comparison and correlations with neuropsychological metrics) we performed a multiple comparisons correction using a False Discovery Rate (FDR) set at 10%. When effects were significant, we explored how robust these effects were by applying more stringent thresholds, including a lower FDR q-value (either 5% or 1%), or a Family-Wise Error (FWE) rate. We report the most conservative multiple comparisons correction (either FDR or FWE) still producing significant effects here.

## Results

We hypothesize that congenital absence of the corpus callosum will alter interhemispheric functional connectivity throughout the cortex. Furthermore, given our prior findings of reduced volume and FA of the ventral cingulum in AgCC individuals [39], we also hypothesize that specific regions are likely to have reductions in both inter- and intrahemispheric connectivity. To address these hypotheses, we estimated global functional connectivity in each subject by averaging across all imaginary coherence measures, producing statistical maps representing each voxel's mean connectivity to all other voxels in the volume [41].

### Resting-State Global Functional Connectivity Comparison: Alpha Band

**Figure 2:**
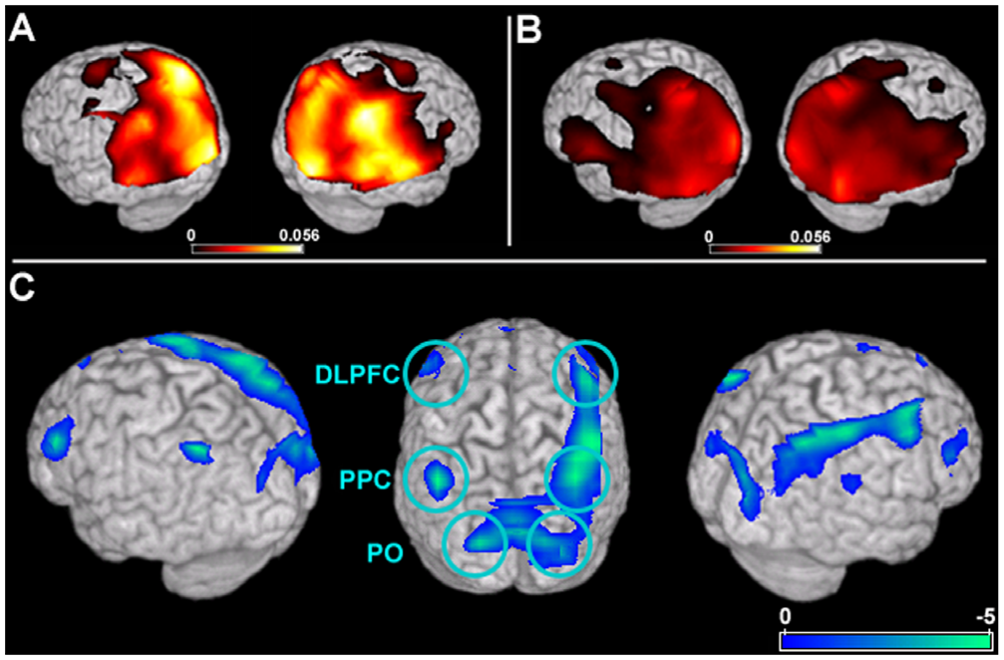
Alpha band (8–12Hz) global connectivity (GC) maps for the HC and AgCC groups and results from a direct comparison between the two samples. In both HC (A) and AgCC (B) groups, high resting-state alpha GC is seen across the occipital, parietal and temporal lobes. In the group contrast, three regions are identified as being significantly underconnected across both hemispheres in the patient group. GC within a region of dorsolateral pre-frontal cortex bilaterally (DLPFC; in blue) is lower in the AgCC group. A cluster of voxels in posterior parietal cortex (PPC; in blue) are significantly underconnected in both hemispheres of patients with AgCC. Caudal to PPC, a region of parieto-occipital cortex (PO) was also disconnected in both hemispheres in the AgCC group. Statistical maps are thresholded (p<0.05, FWE corrected) and superimposed over a rendering of the MNI template brain.

In the alpha (8–12 Hz) range, greatest global connectivity values were present across the same regions of parietal, temporal and occipital cortices in both the AgCC and HC groups (Figure 2A–B). Significant differences in functional connectivity (p<0.05, FWE corrected) between the two groups are shown in Figure 2C. Three regions of cortex were highly underconnected bilaterally (in both the left and right hemispheres) in the patient group (Table 3): the dorsolateral pre-frontal cortex (DLPFC), the posterior parietal cortex (PPC), and the parieto-occipital (PO) sulcus.

**Table 3:** Stereotactic coordinates, locations and abbreviations for cortical regions of interest.

Region	Abbrev	Hemi	BA	x	Y	z	p<(FWE)
***Whole Brain Group Comparison***							
Dorsolateral Pre-Frontal Cortex	DLPFC	Left	46	−55	35	30	0.05
Dorsolateral Pre-Frontal Cortex	DLPFC	Right	46	55	30	20	0.05
Posterior Parietal Cortex	PPC	Left	40	45	−60	50	0.01
Posterior Parietal Cortex	PPC	Right	7	40	−60	50	0.01
Parieto-Occipital Cortex	PO	Left	19	30	−95	20	0.05
Parieto-Occipital Cortex	PO	Right	19	−20	−90	25	0.05
Superior Temporal Gyrus	STG	Right	13	55	−35	20	0.05
Medial Frontal Gyrus	MFG	Right	6	55	5	45	0.01

Region	Abbrev	Hemi	BA	x	y	z	FDR
***Correlation (Verbal Processing Speed)***							
Middle Temporal Gyrus	MTG	Left	22	−45	−20	−10	5%
Superior Temporal Gyrus	STG	Left	22	−55	−40	10	5%
***Correlation (Tower)***							
Medial Pre-Frontal Cortex	MPFC	-	10	15	45	5	0.05
Middle Frontal Gyrus	MFG	Left	8	−20	10	50	0.1

In the frontal lobe, a cluster of voxels over the middle frontal gyri in the left and right dorsolateral prefrontal cortices (DLPFC) were significantly (p<0.01, FWE corrected; Figure 2C) underconnected in participants with AgCC (left mean GC = 0.0176, SD = 0.0071; right mean GC = 0.0322, SD = 0.0082) compared to the HC group (left mean GC = 0.0256, SD = 0.0062; right mean GC = 0.0441, SD = 0.0131). A second region, the posterior parietal cortex (PPC) of both hemispheres, was substantially underconnected (p<0.01, FWE corrected; Figure 2C) in the AgCC cohort (left mean GC = 0.0367, SD = 0.013; right mean GC = 0.0394, SD = 0.0131) compared to healthy controls (left mean GC = 0.052, SD = 0.0081; right mean GC = 0.0548, SD = 0.0078). The third area, a region of the cuneus over the parieto-occipital (PO) sulcus, was bilaterally underconnected (p<0.05, FWE corrected; Figure 2C) in the patient group (left mean GC = 0.0309, SD = 0.0092; right mean GC = 0.0421, SD = 0.0104) compared to healthy controls (left mean GC = 0.0435, SD = 0.0074; right mean GC = 0.0525, SD = 0.0058). In addition, there were two regions that were underconnected unilaterally in the AgCC cohort. In the right hemisphere, a distinct region of the right MFG over Brodmann's Area 6 (p<0.01, FWE corrected; AgCC mean GC = 0.0454, SD = 0.0053; HC mean GC = 0.0543, SD = 0.0074) and a region of the insula (p<0.05, FWE corrected; AgCC mean GC = 0.0324, SD = 0.0076; HC mean GC = 0.0429, SD = 0.0098) were underconnected (Figure 2C, Table 3). Interestingly, there were no overconnected regions in the AgCC group in comparison to matched controls, even at relaxed (uncorrected) statistical thresholds.

### Resting-State Global Functional Connectivity Comparison: Beta/Gamma Band

No significant difference in beta-band imaginary coherence was identified between the two groups (all p>0.05 corrected). Likewise, no significant differences were identified for a group comparison between HC and AgCC for the resting-state gamma reconstructions (all p>0.05 corrected).

### Intrahemispheric and Interhemispheric Connectivity

In AgCC individuals, neuroanatomical changes extend beyond just an impairment or absence of callosal development. In many individuals in this study, the commissural axons that did not cross the midline remained within the same hemisphere, forming Probst bundles [44]. We have also observed reduction in size and fractional anisotropy of ipsilateral tracts, including the cingulum bundle [39]. We therefore wondered whether the observed reductions in resting-state connectivity in the alpha range of these three regions (bilateral frontal (DLPFC) parietal (PPC) and occipital (PO) cortices) are principally the result of compromised callosal development (interhemispheric under-connectivity) or if low resting-state connectivity is also due to impairments in coupling between areas within the hemispheres (intrahemispheric connectivity). To address this issue, we performed a post-hoc evaluation of the hemispheric connections of these three bilaterally underconnected ROIs. For each ROI, total functional connectivity values (mean GC) were averaged across all intrahemispheric and interhemispheric connections separately. These average scores were put into a 2×2×2 ANOVA (group X hemisphere X connection type). This multi-level ANOVA allowed us to examine differences in functional connectivity between HC and AgCC cohorts (group), between ROIs in the left and right hemispheres (hemisphere), and between intrahemispheric and interhemispheric connections of that ROI (connection type).

**Figure 3:**
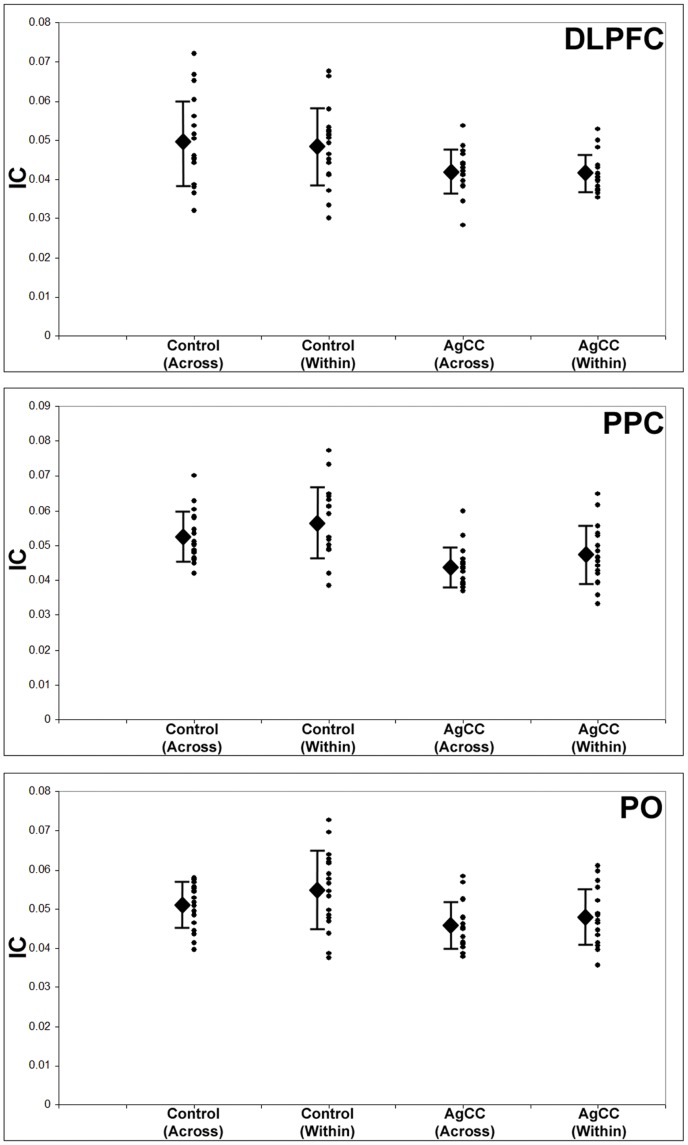
Interhemispheric (across) and intrahemispheric (white bars) connections (averaged GC across all voxels in both hemispheres) for three regions of interest identified as being significantly underconnected in the group contrast, dorsolateral pre-frontal cortex (DLPFC), posterior parietal cortex (PPC) and parieto-occipital cortex (PO). Across the three ROIs, both inter- and intrahemispheric connections are significantly lower in the AgCC group compared to healthy controls. Diamond  =  group mean, error bars  =  S.D. of mean.

For the DLPFC seeds, we were unable to identify a main effect of connection type or hemisphere, and there were no significant two- or three-way interactions (Figure 3A; Table 4). Similarly, also for the PPC and PO seeds, we were unable to identify a main effect of connection type, and no significant two or three-way interactions were identified in our analysis (Figure 3B and 3C; Table 4). In summary, both intra and interhemispheric connections in the alpha range (8–12 Hz) were reduced in the AgCC cohort compared to the HC group, with no significant differences between connection types.

**Table 4:** ROI analysis; between and within-group comparisons.

*Between-Group Pairwise Comparisons*						
ROI	Connection Type	Groups		Df	T	P
DLPFC	Interhemispheric	HC	AgCC	34	2.62	0.013
		HC	pAgCC	25	2.17	0.040
		HC	cAgCC	25	1.89	0.071
		pAgCC	cAgCC	16	0.24	0.815
	Intrahemispheric	HC	AgCC	34	2.69	0.011
		HC	pAgCC	25	2.00	0.057
		HC	cAgCC	25	2.11	0.045
		pAgCC	cAgCC	16	0.44	0.665
PPC	Interhemispheric	HC	AgCC	34	4.03	0.000
		HC	pAgCC	25	3.29	0.003
		HC	cAgCC	25	3.00	0.006
		pAgCC	cAgCC	16		0.923
	Intrahemispheric	HC	AgCC	34	2.87	0.007
		HC	pAgCC	25	2.79	0.010
		HC	cAgCC	25	1.76	0.090
		pAgCC	cAgCC	16	0.10	0.452
PO	Interhemispheric	HC	AgCC	34	2.62	0.013
		HC	pAgCC	25	2.16	0.041
		HC	cAgCC	25	2.10	0.046
		pAgCC	cAgCC	16	0.08	0.935
	Intrahemispheric	HC	AgCC	34	2.38	0.023
		HC	pAgCC	25	2.32	0.029
		HC	cAgCC	25	1.37	0.183
		pAgCC	cAgCC	16	1.05	0.311

*Within-Group Inter/intrahemispheric Pairwise Comparisons*					
ROI		Group	df	T	P
DLPFC		HC	17	0.34	0.739
		AgCC	17	0.24	0.811
		pAgCC	8	0.18	0.862
		cAgCC	8	0.96	0.365
PPC		HC	17	1.38	0.186
		AgCC	17	2.13	0.048
		pAgCC	8	1.33	0.221
		cAgCC	8	1.66	0.135
PO		HC	17	1.82	0.087
		AgCC	17	1.34	0.198
		pAgCC	8	0.26	0.801
		cAgCC	8	1.58	0.153

**Figure 4:**
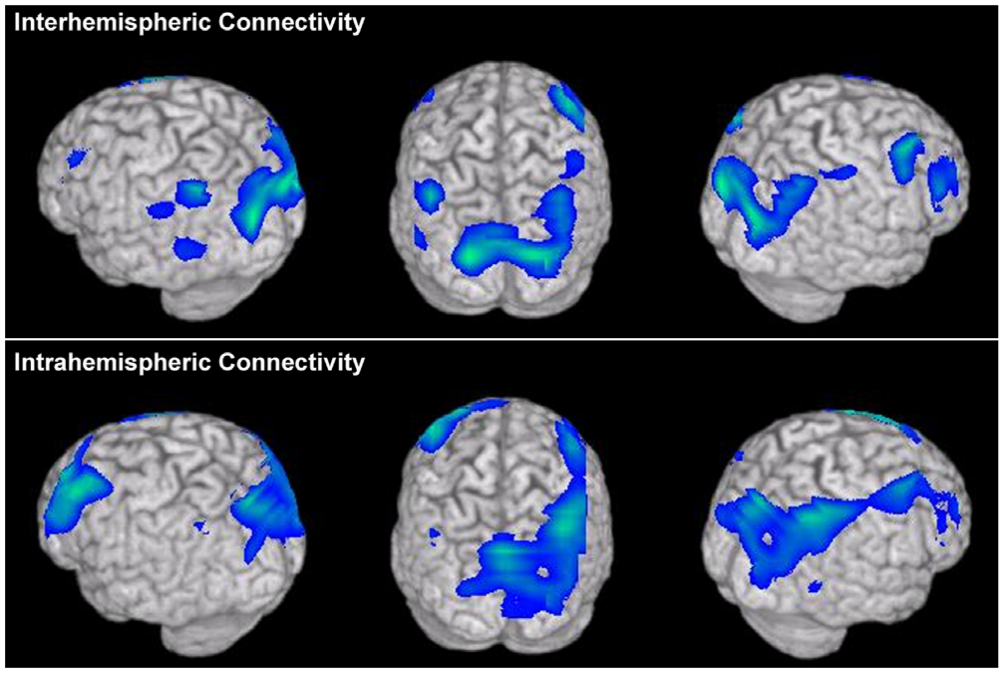
Results from a direct comparison between HC and AgCC groups for connectivity maps split by connection type. For both interhemispheric connections (top row) and intrahemispheric connections of voxels (bottom row), reductions in DLPFC, PPC and PO coherence are predominant bilaterally in the group comparison, similar to the spatial distribution of reduced connectivity observed in the global connectivity maps (Figure 2).

In order to determine if connectivity of cortical fields outside of the three ROI definitions (DLPFC, PPC, PO) were missed in our analysis of intra- and interhemispheric connectivity, we conducted a secondary analysis splitting these two connection types across all voxels. Here, connectivity maps were generated by collapsing across all IC values of a single voxel either with every voxel within the same hemisphere (intra-hemispheric connectivity) or with every voxel in the contralateral hemisphere (inter-hemispheric connectivity). Comparisons between the HC and AgCC groups were then performed using non-parametric unpaired t-tests. For intrahemispheric connections, significant reductions in coherence were observed over DLPFC, PPC and PO bilaterally (Figure 4) with no additional regions showing unique within-hemisphere connectivity. For interhemispheric connections, significant reductions were also observed bilaterally over DLPFC, PPC and PO with no additional regions showing unique cross-hemisphere connectivity (Figure 4). This secondary analysis reproduces what was observed in the ROIs of the 2×2 ANOVA, that reductions in functional connectivity regardless of connection type are present in only these three regions of the brain.

### Planned Comparison: Partial vs. Complete Callosal Agenesis

**Figure 5:**
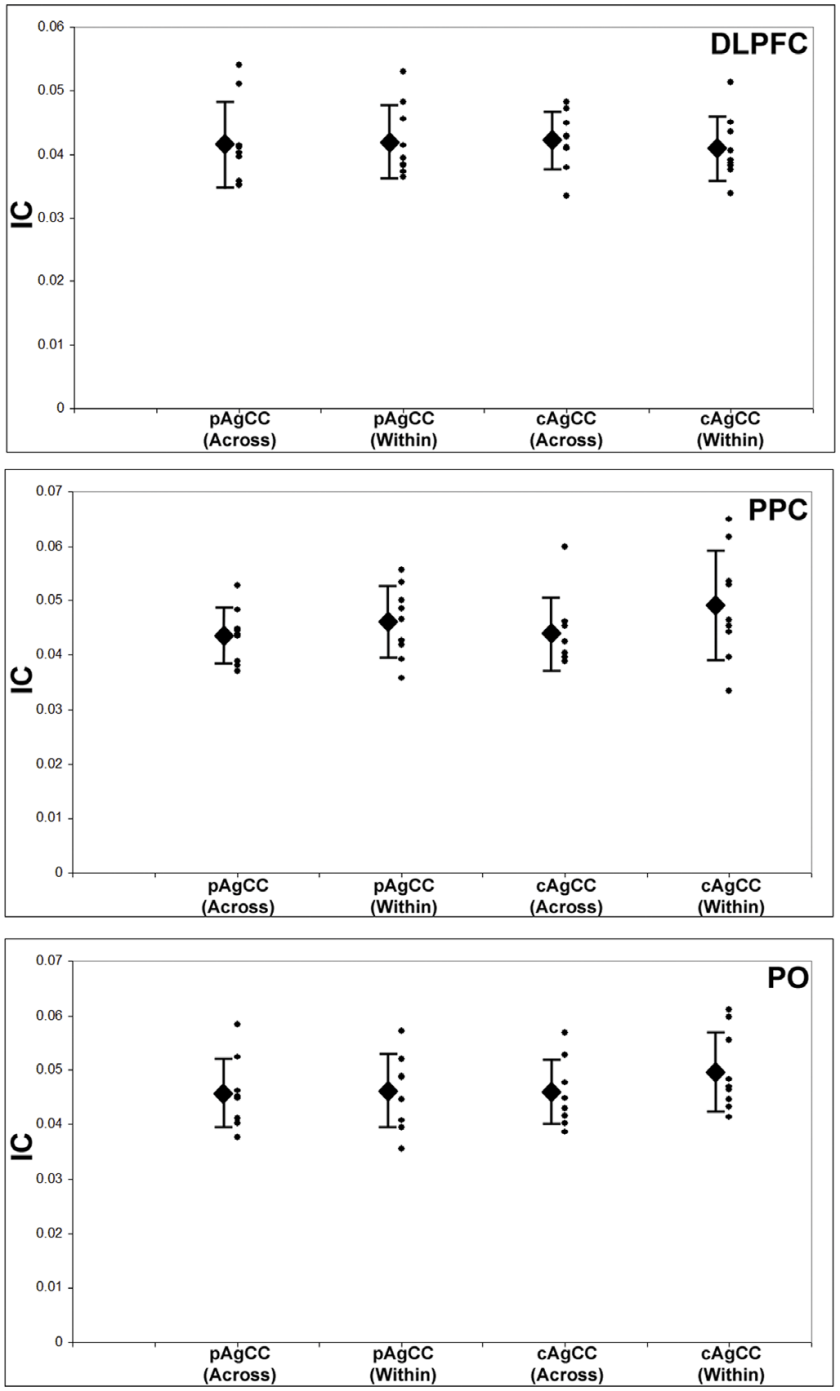
Planned comparison between partial AgCC (pAgCC) and complete AgCC (cAgCC) patients for interhemispheric (across) and intrahemispheric (within) connections of DLPFC, PPC and PO. No significant differences between the pAgCC and cAgCC groups are identifiable in any of the ROIs. Conventions as in Figure 3.

In order to examine potential differences between patients with complete callosal absence (cAgCC) and those with remnant callosal fibers (pAgCC), we also conducted a planned comparison between these two groups for the connection types of each ROI. For intrahemispheric connections, no significant differences (all p>0.05; Table 4, Figure 5) were observed between the two groups for DLPFC (pAgCC mean = 0.0420, SD = 0.0057; cAgCC mean = 0.0409, SD = 0.0051), PPC (pAgCC mean = 0.0460, SD = 0.0066; cAgCC mean = 0.0491, SD = 0.0102), or PO (pAgCC mean = 0.0463, SD = 0.0068; cAgCC mean = 0.0498, SD = 0.0072). For interhemispheric connections, no significant differences (all p>0.05; Table 4) between the two groups were observed for DLPFC (pAgCC mean = 0.0415, SD = 0.0067; cAgCC mean = 0.0422, SD = 0.0046), PPC (pAgCC mean = 0.0436, SD = 0.005; cAgCC mean = 0.0438, SD = 0.0066) or PO (pAgCC mean = 0.0457, SD = 0.0063, cAgCC mean = 0.0459, SD = 0.006).

### Correlations Between Functional Connectivity and D-KEFS Measures

**Figure 6:**
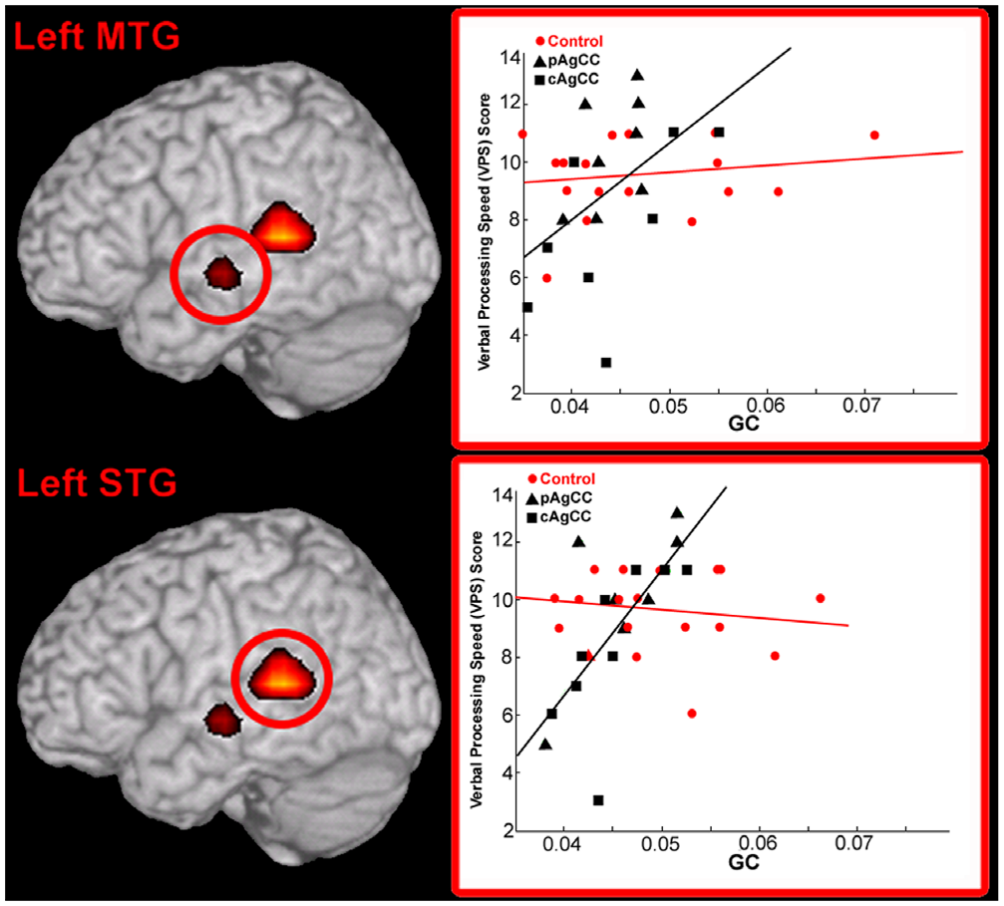
Results of a voxel-wise correlation between global imaginary coherence (GC) at rest in the AgCC cohort (scatterplots, in black) and verbal processing speed (VPS) performance on the D-KEFS Color-Word Interference Test. Functional connections of two regions in the left hemisphere were positively correlated (overlay, in red) with processing speed in AgCC, in the left medial temporal gyrus (MTG) and the superior temporal gyrus (STG). These same two regions were not found to be significantly correlated with processing speed in healthy controls (scatterplots, in red). No regions in the right hemisphere were correlated with verbal processing speed in any group. pAgCC  =  partial agenesis of the corpus callosum. cAgCC  =  complete agenesis of the corpus callosum. Statistical maps (Pearson's *r*) are thresholded (p<0.05, 1% FDR correction) and superimposed as in previous figures.

We hypothesized that the degree of resting-state functional connectivity correlates with cognitive measures of processing speed and problem solving. To test this hypothesis, we performed a correlation between global connectivity values and scaled performance measures from the D-KEFS. In the AgCC cohort, GC of two regions of the left temporal lobe significantly correlated with verbal processing measures (CWIT-VPS; Figure 6, in black). Functional connectivity of Brodmann's Area 22 (BA22; Table 3) within the left middle temporal gyrus (MTG; Figure 6, in black) was positively correlated with faster verbal processing speed (r = 0.744, p<0.05, 5% FDR correction). A similar relationship was identified in a caudal portion of left BA22, in the superior temporal gyrus (STG; Figure 6), where functional connectivity was also positively correlated with faster verbal processing speed (r = 0.801, p<0.05, 5% FDR correction). However, in the control group, no regions showed a significant relationship between resting-state GC values and the CWIT-VPS scores, including the MTG (r = 0.28, p = 0.344; Figure 6, in red) and STG (r = -0.15, p = 0.55, Figure 6, in black). Likewise, when collapsed across the larger combined AgCC and control groups, no significant correlation between VPS scores and global connectivity was identified for either the STG (r = 0.282, p = 0.10) or MTG (r = 0.279, p = 0.10) clusters. In order to evaluate the effect of age and IQ on functional connectivity and VPS scores within the AgCC cohort, we computed separate partial correlation coefficients for both MTG and STG. Correlations between GC values and VPS scores remained statistically significant for both regions when correcting for age (MTG, r = 0.700, p = 0.003; STG, r = 0.709, p = 0.002) and IQ (MTG, r = 0.652, p = 0.006; STG, r = 0.594, p = 0.02). These two regions—both correlated to verbal processing speed—were only in the left hemisphere and were spatially distinct from each other, with no overlap between the clusters in the MTG and the STG at a 5% FDR corrected threshold in the AgCC group alone.

**Figure 7:**
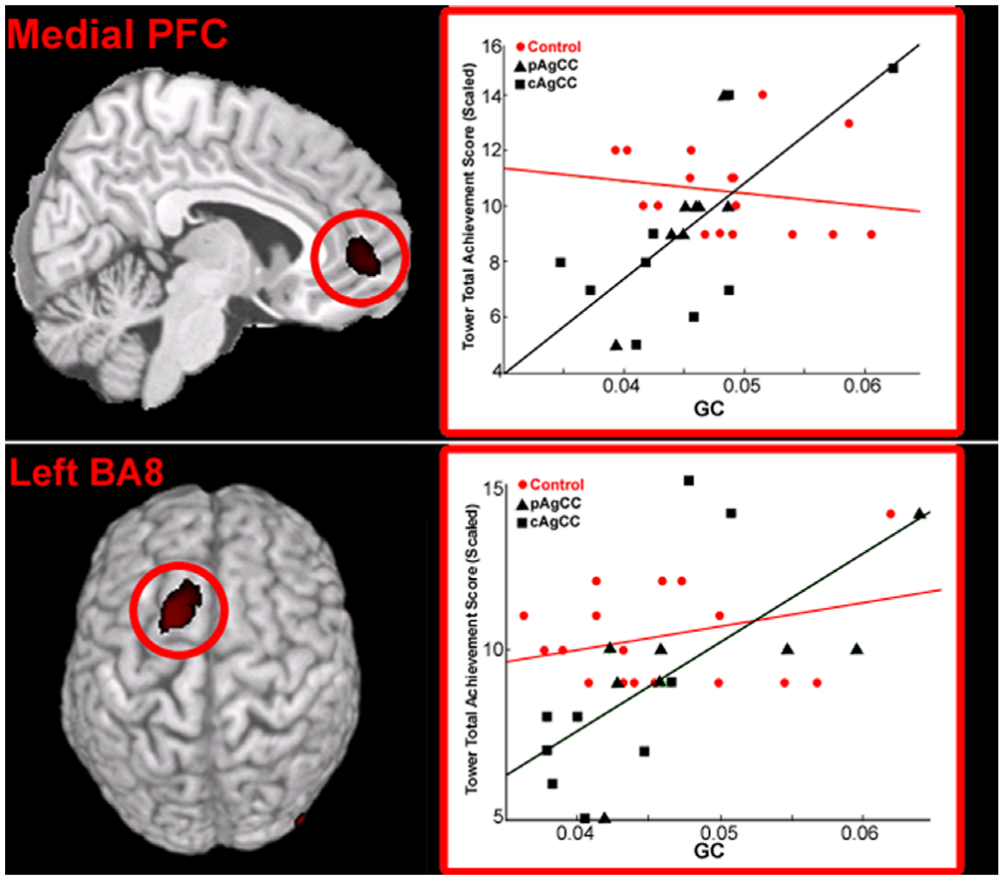
Results of a voxel-wise correlation between global imaginary coherence (GC) at rest in the AgCC cohort (in black) and problem solving (PS) performance on the D-KEFS Tower Test. Functional connectivity of a region in medial pre-frontal cortex (PFC, top panel) and the left superior frontal gyrus in Brodmann's Area 8 (BA8; bottom panel) was positively correlated with Tower scores (p<0.05, 5% FDR correction) in the AgCC group. No significant relationship between performance on Tower and functional connectivity was identified in the healthy control group (in red). Conventions as in Figure 4. Statistical maps (Pearson's r) are thresholded (p<0.05, 10% FDR correction) and superimposed as in previous figures.

When global imaginary coherence values were compared with the Total Achievement scores from the Tower Test (TT-TA), functional connectivity of two regions in pre-frontal cortex (PFC) positively correlated with TT-TA scores in individuals with AgCC. The first area, in an anterior region of medial PFC (mPFC; Table 3) was positively correlated (r = 0.701, p<0.05, 5% FDR correction) with TT-TA scores (Figure 7). A second region, within the left PFC (BA8; Table 3) was also significantly correlated (r = 0.665, p<0.05, 10% FDR correction) with TT-TA performance (Figure 7). In the healthy control group alone, no regions were identified as having a significant correlation between functional connectivity and TT-TA scores (Figure 7, in red). However, when analyzing the data collapsed across both AgCC and HC groups, a significant correlation between TT-TA and global connectivity scores of mPFC remains (r = 0.426, p<0.015). In the healthy control cohort, a weak positive trend was seen (p = 0.07) between functional connectivity of a cluster of voxels in PFC (neighboring the region of BA8 where a positive correlation was identified in the AgCC group) and TT-TA scores becomes statistically significant when collapsed across both HC and AgCC groups (r = 0.515, p<0.005, uncorrected). Using a partial correlation coefficient, correlations between GC and TT-TA scores remain statistically significant when corrected for age (BA8, r = 0.682, p = 0.004; MPFC, r = 0.706, p = 0.022) and IQ (BA8, r = 0.645, p = 0.007; MPFC, r = 0.686, p = 0.003) in the AgCC cohort.

## Discussion

This study provides compelling evidence that the absence of a fully developed corpus callosum impacts functional interactions both within each hemisphere and between the two hemispheres in the resting alpha frequency range (8–12 Hz). This impairment of functional interactions appears to be most evident in regions within the frontal, parietal and occipital cortices that are implicated in cognitive domains known to be impaired in individuals with AgCC. We also found that the degree of diminished connectivity in specific cortical regions was directly correlated with verbal processing speed and executive performance in these individuals. In our sample, there was substantial anatomical (e.g. pAgCC, cAgCC, Probst bundles) and behavioral (processing speed and/or problem solving impairment) variability, similar to what has been identified in the general AgCC population [23]. Even within this range of anatomical impairment, the same regions of frontal, parietal and occipital cortex were underconnected in the AgCC cohort, and a broad range of cognitive performance in the AgCC participants allowed us to identify relationships between connectivity and executive function.

### Reductions in anatomical and functional connectivity in callosal agenesis

The posterior resting-state alpha network itself is not grossly redistributed in individuals with AgCC (Figure 2). Instead, in the absence of developing this major white matter tract, we observed decoupling between cortical fields that act as key hubs of integration and cognitive processing. For example, the most anterior region to show functional underconnection in the AgCC cohort, DLPFC, plays a primary role in executive function [38], [57]. Individuals with lesions of the DLPFC show deficits in maintaining a rule set and task switching [58]–[62]. Similarly, individuals with callosal agenesis show impairments in abstract problem solving, categorization, and task switching [31]–[32], [61]. The second region to show reduced functional connectivity in AgCC, PPC, is involved in navigating an attentional “spotlight” across sensory receptive fields [63]–[65]. Patients with AgCC show difficulty in switching between spatially cued locations [66] and exhibit difficulties in both spatial navigation (maze-solving) and visually-based somatosensory discrimination (tactile foam board; [67], [68]). The third region underconnected in the AgCC cohort, area PO, also plays a primary role in visual attention [69] and reductions in alpha coherence over PO could lead to deficits in awareness and visual attention in patients with AgCC [66]. Future investigations are needed in order to elucidate how reductions in functional connnectivity in AgCC directly contribute to deficits of task-switching and spatial attention.

Previous studies using fMRI or EEG have suggested decreased connectivity in individuals with AgCC [70]–[74]. However, these studies have not benefited from a large sample size (n>15) or a control cohort properly matched for age and IQ. An fMRI case study of three individuals with AgCC demonstrated reduced functional connectivity in regions serving auditory, motor and language processing, although with such a small sample size, the statistical significance of this reduced connectivity was difficult to determine [73]. In EEG, reductions in resting-state alpha coherence have been repeatedly reported in individuals with AgCC, generally between frontal and central scalp electrodes [72], [75]–[76]. The precision of source localization inherent to MEG-I analyses used in the present study allows us to target which brain areas are specifically underconnected in callosal dysgenesis, a step far beyond the limitations of EEG spatial resolution. Reductions in frontal and central alpha oscillatory coupling previously reported in EEG are likely due to impairments in functional connectivity specific to DLPFC, PPC and PO bilaterally. With careful participant matching, we can exclude that this change in MEG-I coherence is due to an age or general intelligence group difference.

While previous studies have suggested that, in agenesis of the corpus callosum, interhemispheric interactions are severed while intrahemispheric connections are relatively spared (or in some cases enhanced; see [77]) existing data addressing this question are limited and contradictory. Reduced interhemispheric transmission during behavior in AgCC (such as the propagation of visual evoked potentials between the two hemispheres) has been previously characterized [78], [79]. We find no evidence of exclusive reductions in interhemispheric connectivity in AgCC (Figure 4), as both within and cross-hemisphere connections of parietal, frontal and occipital cortex are lower in patients with either complete or partial callosal agenesis. It is unclear how the remnant segments of the corpus callosum in pAgCC or concomitant anatomical changes in cAgCC contribute to a preservation of functional connectivity in this disorder. Further investigation is necessary in order to understand how these concomitant anatomical changes (including enlargement of the anterior commissure) contribute to deficiencies in core abilities such as language.

### Functional significance of coherence in cortical oscillations

Our main findings are specific to the alpha frequency range which is prominent in MEG-I power analyses and can be modulated by levels of awareness, representing a true “resting-state” frequency in the brain. Posterior alpha is associated with the maintenance of “idling” activity within visual thalamocortical generators during an alert, wakeful state [80]–[83]. During spontaneous EEG/MEG recordings, dipoles that localize to the frontal lobe have also been identified [84]. Alpha power is modulated by levels of awareness and visual attention, analogous to resting-state networks identified in fMRI [85]. It could be argued that the changes in functional connectivity we observe in our dataset are confounded by a fundamental difference in the mental states present between any clinical group and controls during a resting period (see [86] for a discussion of this issue). A similar issue has been raised in the discussion of interpreting resting-state fMRI data [87] although both spontaneous fMRI and MEG data is known to be highly reliable, reproducible and state-independent [88]–[90].

While coherent networks in the beta (12–30 Hz) and gamma (30–55 Hz) frequency bands were reconstructed and identified in both the HC and AgCC groups, no significant group differences in functional connectivity were found in either frequency band. Although some signal is present in the resting-state MEG recordings in these higher frequency bands (Figure 1), it is challenging to consistently identify robust power in these ranges. Furthermore, within-session reliability was ranked low for the theta, beta and gamma bands when compared to ICC scores for the alpha band. It is possible that an absence of an identifiable group difference in frequency ranges other than alpha is merely due to low sensitivity for these resting-state functional connectivity reconstructions from other frequency bands.

### Functional connectivity and cognitive abilities

The examination of spontaneous brain activity and, more specifically, interregional interactions using neuroimaging has recently emerged as a powerful research approach in determining both standard and pathological brain states, as it does not rely on patient compliance or the need to average data across multiple trials for sufficient power [91]–[93]. More importantly, it has been recently determined that these resting-state cortical networks are not only highly reliable and reproducible, but that correlations in these networks are directly related to behavioral abilities assessed outside of the scan session. In EEG, resting-state coherence between scalp electrodes is related to problem solving abilities and functional outcomes measured in standard behavioral tests [94], [95]. A similar relationship has been identified in spontaneous fMRI studies, where compromised cognitive strategies due to aging are related to degradations in connectivity within classic resting-state networks [96]. Furthermore, cognitive training paradigms can modulate cortical resting-state interactions identified using positron emission tomography [97].

Poor performance on both tests of verbal processing speed and problem solving was found to be related to low levels of functional connectivity in specific regions of the brain. For the verbal processing speed score, two regions of the left temporal lobe (MTG, STG) were positively and significantly correlated with this measure (Figure 6). These regions are primary components of a speech reception and production network, particularly within a putative dorsal processing stream in the left hemisphere serving sensory-motor transformations during vocalization [98]. An absence 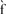 a correlation between functional connectivity and performance of these areas in healthy controls suggests that connectivity from these regions may represent a selective vulnerability in AgCC individuals. It will be interesting to address how this impairment in resting-state functional connectivity of the left temporal lobe impacts cortical oscillations during language production itself, in AgCC and other neurological disorders. A somewhat different relationship was seen in pre-frontal cortex between resting-state connectivity and performance on the Tower test. In the patient group, global IC scores of left DLPFC and medial PFC strongly correlated with tower scores (Figure 7). While not statistically significant, a trend between functional connectivity and tower scores was identified in the control group as well, particularly within the left DLPFC. Unlike the correlations between left temporal lobe functional connectivity and verbal processing speed scores mentioned above, it is possible that the resting-state functional connectivity of PFC represents a generalized relationship of alpha coherence of left DLPFC and problem solving ability, independent of disease state.

It is also possible that concomitant anatomical tracts that develop specifically in agenesis patients such as Probst and heterotopic bundles [99], [100] influence inter- and intrahemispheric connectivity and related underlying cognitive mechanisms. Although we are unable to determine an increase in alpha intrahemispheric connectivity for any region with reduced functional connectivity in the AgCC group, given the heterogeneity of anatomical malformations present in both cAgCC (Table 1) and pAgCC groups (Table 2) it remains a possibility that such tracts could act as compensatory mechanisms preserving performance in these tasks. In fact, the Probst and heterotopic callosal bundles have been posited to be anatomical structures that aid in intra- and interhemispheric transfer during cognition, although this has yet to be shown in a group matched across these abnormalities with sufficient sample size [101], [102]. Regardless, full development of the corpus callosum (including fibers that project to and emanate from DLPFC) may be necessary for an individual to learn problem solving strategies. Indeed, in neurodevelopmental disorders of connectivity like autism where interhemispheric interactions are evident [40], callosal integrity is known to correlate with performance on the Tower test [103], [104].

### Conclusions

In conclusion, MEG-I can be used to identify how alterations in neuroanatomical connectivity (e.g. callosal fibers in AgCC) translate into faulty functional coupling in a discrete frequency range (alpha; 8–12 Hz) both between and within the cerebral hemispheres, even within the brain's “resting state”. In AgCC, the degree of disconnection that occurs when callosal fibers fail to develop is directly related to processing speed and problem solving, indicating that these measures represent real-time brain interactions. These findings also serve to validate MEG-I as a tool to understand the impact of compromised cortical interactions on essential cognitive abilities [42], [105], [106].
